# Interactions of metabolic syndrome and thyroid hormones

**DOI:** 10.1186/1756-6614-6-S2-A9

**Published:** 2013-04-05

**Authors:** Georg Brabant

**Affiliations:** 1 Experimental and Clinical Endocrinology, MK I, University of Lübeck, Germany

## 

The prevalence of metabolic syndrome worldwide is high and rising (Alexander Diabetes 52: 1210, 2003; Cameron Endocrinol Metab Clin North Am 33: 351, 2004). According to all definitions visceral obesity, increased triglycerides, decreased HDL cholesterol, impaired glucose tolerance and increased blood pressure are part of the definition. Thyroid hormones impact on all these factors. In a large Chinese case control study all components of the metabolic syndrome were associated with systematically higher TSH levels (Lai Endocr J 58:23, 2011). Recent data on TH replacement following thyroidectomy further strengthen the close relation between TH status and body weight. Total thyroidectomy despite full replacement with T4 increases body weight by a mean of 2 kg above controls within 1 year (Jonklaas Thyroid 2011). When patients are treated with T3 instead of T4 to an identical 24 h mean of TSH body weight decreases by more than 2% within 1 year (Celi JCEM 96: 3466, 2011). The substantial effects of thyroid hormones on lipid components have been frequently reviewed (Duntas Med Clin North Am. 2012). Thyroid hormones are positively associated with insulin resistance not only in clinically diagnosed DM but also in subjects with a normal glucose tolerance. Indices of insulin resistance as judged by HOMA (which assesses fasting and postprandial insulin resistance) are closely linked to TH status. They modulate gluconeogenesis and peripheral glucose uptake (Duntas, Orgiazzi, Brabant Clin Endocrinol 2011). Finally, recent data from children and adolescents show a major impact of TH on the regulation of blood pressure. As other components of blood pressure dysregulation are less prominent at this age the small positive association of thyroid hormones with systolic and diastolic blood pressure support previous studies on the impact of TH status on blood pressure regulation (Ittermann JCEM 97: 828, 2012). Thus, all components of the metabolic syndrome are altered by thyroid hormone status. Measurement of thyroid hormone status ought thus to be considered in any patient with metabolic syndrome or diabetes mellitus to rule out an easily manageable cofactor of the disease.

